# Epidemiology and prognosis in young lung cancer patients aged under 45 years old in northern China

**DOI:** 10.1038/s41598-021-86203-4

**Published:** 2021-03-25

**Authors:** Jin Shi, Daojuan Li, Di Liang, Yutong He

**Affiliations:** Cancer Institute, The Fourth Hospital of Hebei Medical University, The Tumor Hospital of Hebei Province, Shijiazhuang, 050011 Hebei People’s Republic of China

**Keywords:** Cancer, Biomarkers, Health care, Oncology, Risk factors

## Abstract

To explore the epidemiological characteristics and prognosis of lung cancer in patients aged under 45 years old in northern China. The population-based database about lung cancer cases aged under 45y selected form the Hebei Provincial Cancer Registry Center from 2010–2015. Mortality data of young death from 1973 to 1975, 1990 to 1992, and 2004 to 2005 were extracted from the national retrospective survey of death. Mortality rates were calculated by the mortality database above in this analysis. Consecutive, 954 non-selected younger patients (< 45 years) and 2261 selected older patients (≥ 45 years) with pathologically diagnosed lung cancer treated at the Fourth Hospital of Hebei Medical University were included as the hospital-based database. Epidemiological, treatment outcomes and prognosis status from 2010 to 2017 were documented. A comparison with younger and older patients was also made. Multivariate analysis with young lung cancer patients was calculated by Cox regression model. The younger lung cancer mortality rate tended to slightly increase in Hebei Province, from 1.04 per100 000 in 1973 to 2.01 per 100 000 in 2015, but the PDR tended to decrease over the last 40 years. There were 954 younger and 2261 older lung cancer patients included in the hospital-based database. The proportions of patients who were female (50.84% vs 34.85%), family history of cancer (12.37% vs 6.32%), advanced stage at diagnosis (65.46% vs 60.77%) and adenocarcinoma (65.27% vs 61.11%) were relatively higher in the younger group than in the older group. The median OS were 23.0 months and 27.0 months between younger and older, the OS difference existed between the two groups (*P* = 0.001). In the younger patients, Cox regression showed that a family history of cancer, symptoms at diagnosis, pathology, stage at diagnosis and surgery were confirmed as independent factors affecting the prognosis. Mortality rates among younger lung cancer patients showed an increasing trend in northern China. The younger account for small but have unique characteristics, with higher proportions of female, family history of cancer, adenocarcinoma and advanced stages than the older group and had a trend of worse OS.

## Introduction

Lung cancer, which has a variety of histological subtypes, is a multi-step and multi-factorial disease. According to GLOBOCAN 2018, there were approximately 2.1 million new lung cancer cases and 1.8 million lung cancer-related deaths worldwide^1^. In China, lung cancer is the most common cancer and a major cause of cancer-related death disease, and in the last ten years, the incidence rate has increased 12.16%^2,3^. Although an analysis of 17 population-based registries showed that the survival rate in lung cancer patients had increased from 16.1% in 2003–2005 to 19.7% in 2012–2015, the prognosis remains very poor, and lung cancer poses a serious threat to human health in China^4^. Hebei Province, located in northern China, has one of the highest incidence rates of lung cancer, with an incidence rate that is 1.10-fold than the national rate^5,6^. Lung cancer is predominantly identified in the relatively older population; therefore, several international organizations have suggested that to discover more cases of lung cancer in the early stage, when they could be cured, the age at which lung cancer screening should start is 55–74 years in the USA and 50–74 years in China^7–9^. However, the smaller but not insignificant proportion of younger patients should not be ignored.

Lung cancer is relatively rare in the younger population, and less than 3.5% of lung cancer patients are younger than 45 years old^10^. However, in recent decades, as the incidence of lung cancer has increased, the average age at diagnosis has decreased^11^. Lung cancer in young patients is a poorly studied clinical entity, and only a few studies have described the risk factors for and clinical characteristics, incidence, mortality and outcomes of lung cancer in this population^12,13^. In addition, the results of previous research on lung cancer in younger patients are not completely consistent. On the one hand, the results consistently suggested that lung cancer cases in young patients may constitute a clinical entity with distinct clinical and pathological characteristics, with fewer patients who were smokers, more female patients, a predominance of adenocarcinoma and a more advanced stage at initial diagnosis^13–15^. On the other hand, the proportions of patients with mutations in the epidermal growth factor receptor (EGFR), a family history of cancer and a poor prognosis differed among reports^11,16–24^. Besides that, carcinoembryonic antigen (CEA), squamous cell carcinoma antigen (SCC) and neuron-specific enolase (NSE) are common biomarkers that are used to diagnose lung cancer and make and prognostic predictions, but the differences in biomarker levels among lung cancer patients stratified by age have not yet been reported^25,26^.

To our knowledge, few large-scale population- or hospital-based studies involving younger Chinese lung cancer patients and assessing detailed data on epidemiological, clinical and prognostic factors have been conducted. In this study, we analysed data on younger lung cancer patients from the national retrospective survey of mortality, a population-based cancer registry and a hospital-based database of lung cancer cases in young patients in Hebei Province. Because of the distinct biological behaviour of lung cancer in the younger population, limited number of previous studies and conflicting results, there was a pressing need to conduct this study to confirm the epidemiological and clinical characteristics by analysing the previously controversial factors and assessing the factors not yet investigated in this population.

## Materials and methods

### Data source

#### National retrospective survey of mortality

In the mid-1970s, a nationwide retrospective survey of the causes of mortality of 56 diseases (with a special emphasis on cancer), organized by the National Office for Cancer Prevention and Control, was conducted in 29 provinces, including Hebei Province. This survey covered all 153 cities and counties in Hebei Province. For the first time, it provided the national profiles and patterns of cancer mortality, as well as the total mortality rate in Hebei Province^27^.

A national retrospective sampling survey of cancer mortality was organized in 1990–1992 by the National Office for Cancer Prevention and Control. This survey employed a stratified sampling method and covered approximately 10% of the Chinese. 21 regions in Hebei Province were enrolled^28^.

A retrospective survey of all death causes for the period was organized in China, 2004–2005. It was carried out in 31 provinces/municipalities/autonomous regions, including Hebei Province in China in 2006. A total of 18 cities and counties were selected as sampling areas, with a total population of 13,791,868 persons (20.2% of the total population of Hebei Province)^29^.

#### Population-based cancer registration data

The Hebei Provincial Cancer Registry Centre was established in 2009 and became the primary entity responsible for data collection starting in 2010. The latest data collected were from 2015. A total of 33 population-based cancer registries in Hebei Province submitted data from 2010 to 2015, and the data from 21 population-based cancer registries qualified for inclusion in the pooled data used in the final analysis. These cancer registries covered 50.28 million person-years, accounting for 11.5% of the population in Hebei Province^30^.

#### Hospital-based young lung cancer cases

The Fourth Hospital of Hebei Medical University, also known as the Tumour Hospital of Hebei, is one of the largest and most comprehensive hospitals in Hebei. We collected all the data pertaining to 954 young patients diagnosed with lung cancer at the Fourth Hospital of Hebei Medical University between 1 January 2010 and 31 December 2017. Patients older than 45 years who were diagnosed with lung cancer were sampled as follows: those meeting the inclusion criteria were stratified by the month in which they were diagnosed, and the patients in each month were divided into ten groups based on a random number table. There were no significant differences in sex or age among the ten groups (P > 0.05). Then, we selected one number randomly from each group for inclusion in the analysis. Eventually, 2261 lung cancer patients older than 45 years were enrolled in the study. Age, sex, family history of cancer, smoking history, drinking history, height, weight, onset symptoms, tumour location, cancer stage at diagnosis, pathology, serum biomarkers, EGFR mutation status, therapies and survival status were collected in detail.

### Ethics statement

The use of the data from the lung cancer patients in the population-based and hospital-based cancer registries was approved by the Ethics Informed Committee of the Fourth Hospital of Hebei Medical University (Shijiazhuang, Hebei, China), and all analyses were performed in accordance with the approved guidelines. Written informed consent was obtained from all subjects or from their next of kin if the patients were deceased.

### Follow-up

The date of death or the last follow-up date if the subject was still alive were also collected. The last follow-up date was December 31st, 2019. Overall survival (OS) was defined as the time from diagnosis to death from any cause, and patients who remained alive were censored at the end of the study. Censored subjects included patients who were lost to follow-up and those who had not experienced the outcome (death) before the end of the study period.

### Statistical analysis

Using the data from the population-based database, the CMR (crude mortality rate), ASRW (age-standardized rate using the world standard population), ASRC (age-standardized rate using the China standard population (2000)), and PDR (proportional death rate) were calculated with SAS software.

Using the data from the hospital-based database, continuous variables were summarized as the means with standard deviations or medians with ranges; categorical variables were summarized as frequencies and percentages. Pearson’s chi-squared test was used to assess the significance of differences in categorical variables between the younger and the older. A Kaplan–Meier analysis was performed. Multivariable analyses were performed with a Cox proportional hazards model. All analyses were conducted using the SPSS for the Windows sofware system (version 21.0; IBM Corp, Armonk, NY, USA) (https://www.ibm.com/support/pages/node/213045) and the “survminer” and “forestplot” package inthe software R3.1.3 (R Core Team, 2015) (https://mirrors.tuna.tsinghua.edu.cn/CRAN/). Statistical significance was determined by a two-tailed p value ≤ 0.05. Any missing values were reported as unknown, and thus, not all the study population was included in the different analyses.

## Results

### Trend in mortality trend among young lung cancer patients in Hebei Province in the population- based database, 1973–2015

**Figure 1:**
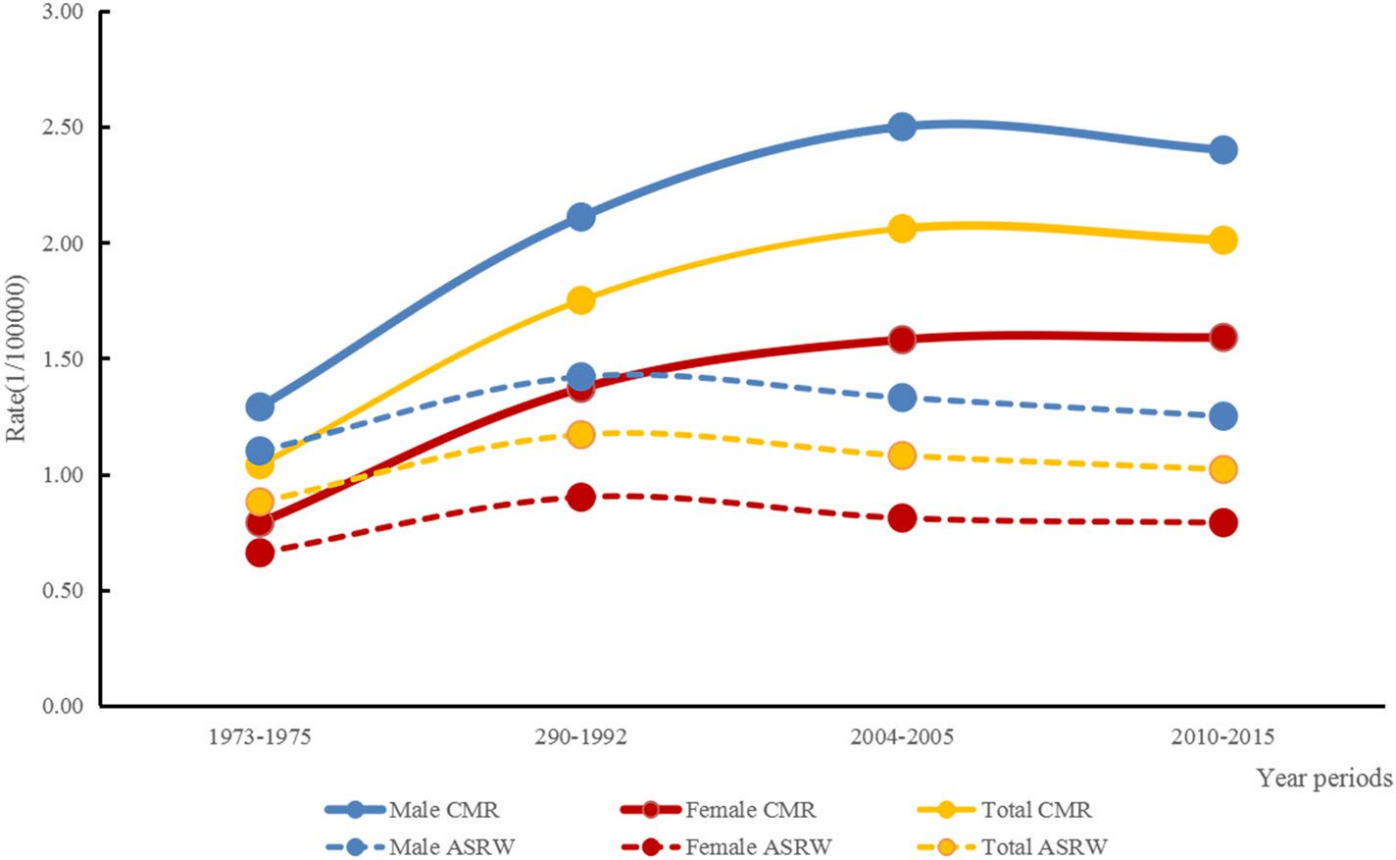
Mortality trend of younger lung cancer in Hebei Province by population-based database, 1973–2015.

As shown in Table 1 and Fig. 1, the lung cancer mortality rate in younger tended to slightly increase in Hebei Province from 1973 to 2015. In 1973–1975, the CMR was 1.04 per 100,000 (1.29 per 100,000 for males and 0.79 per 100,000 for females). It increased to 2.01 per 100,000 (2.40 and 1.59 per 100,000 for males and females) in 2010–2015. In 1973–1975, the ASRW in younger was 0.88 per 100,000, accounting for 8.73% of all lung cancer-related deaths. In 1990–1992, it was 1.17 per 100,000, accounting for 7.14%. The lung cancer ASRW was 1.08 per 100,000, with a PDR of 4.84% in 2004–2005. In 2010–2015, the ASRW was 1.02 per 100 000, with a PDR of 3.40%. The ASRW in 2010–2015 was 93.27% higher than in 1973–1975. During the study period, the ASRW increased in both men and women; in contrast, the PDR tended to decrease over the last 40 years, with 63.15% and 56.84% reductions in males and females, respectively. The PDR was higher in females than in males. In addition, the median age at death of young lung cancer patients was 38.80 years in 1973–1975, 38.77 years in 1990–1992, 39.44 years in 2004–2005 and 40.83 years in 2010–2015.

**Table 1:** Younger lung cancer mortality in Hebei Province from 1973 to 2015.

	1973–1975			1990–1992			2004–2005			2010–2015		
	Total	Male	Female	Total	Male	Female	Total	Male	Female	Total	Male	Female
CMR	1.04	1.29	0.79	1.75	2.11	1.37	2.06	2.50	1.58	2.01	2.40	1.59
ASRC	1.11	1.38	0.83	1.46	1.77	1.14	1.35	1.66	1.03	1.23	1.48	0.97
ASRW	0.88	1.10	0.66	1.17	1.42	0.90	1.08	1.33	0.81	1.02	1.25	0.79
PDR	8.73	8.54	9.08	7.14	6.71	7.98	4.84	4.61	5.30	3.40	3.15	3.92

*ASRC* age-standardized rate using China standard population (2000), *ASRW* age-standardized rate using World standard population, *CMR* crude mortality rate, *PDR* proportional death rate = younger lung cancer deaths**/**whole lung cancer deaths*100%).

### Characteristics of lung cancer patients in the hospital-based database

In total, 954 non-selected younger patients and 2261 randomly selected older patients were from the database of the Tumour Hospital of Hebei Province from 2010 to 2017 for inclusion in this study. Patient characteristics in the younger and older groups are shown in Table 2.

**Table 2:** The characteristics of lung cancer patients by hospital-based database.

Items	≤ 45 (N = 954)			> 45 (N = 2261)				
	Total	N	Pro (%)	Total	N	Pro (%)	X^2^	*P*
**Gender**	954			2261				
Male		469	49.16		1473	65.15		
Female		485	50.84		788	34.85	71.692	**0.000**
**Family history**	954			2261				
Yes		118	12.37		143	6.32		
No		836	87.63		2118	93.68	32.861	**0.000**
**Smoking**	954			2261				
Yes		275	28.83		1120	49.54		
No		679	71.17		1141	50.46	117.146	**0.000**
**Drinking**	954			2261				
Yes		290	30.40		933	41.26		
No		663	69.50		1328	58.74	32.96	**0.000**
**BMI**	940			2211				
< 24.0		499	53.09		1167	52.78		
> 24.0		441	46.91		1044	47.22	0.024	0.876
**Symptoms at diagnosis**	954			2261				
Yes		773	81.03		1979	87.53		
No		181	18.97		282	12.47	22.997	**0.000**
**Side**	906			2233				
Left		390	40.88		1012	44.88		
Right		516	54.82		1221	54.18	1.348	0.246
**Subside**	534			1556				
upper lobe		255	47.75		823	52.55		
Middle lobe		75	14.04		181	11.56	4.74	0.090
Low lobe		204	38.20		552	35.25		
**Stage at diagnosis**	718			1695				
I + II		311	43.31		665	39.23		
III +I V		470	65.46		1030	60.77	68.14	**0.000**
**Pathology**	766			1764				
Adenocarcinoma		500	65.27		1078	61.11		
Squamous-cell carcinoma		77	10.05		434	24.60		
Small cell carcinoma		189	24.67		252	14.29	90.535	**0.000**
**CEA**	680			1185				
Normal		387	56.91		685	57.81		
Abmormal		293	43.09		500	42.19	0.14	0.707
**SCC**	469			866				
Normal		397	84.65		728	84.06		
Abmormal		72	15.35		138	15.94	0.08	0.780
**NSE**	649			793				
Normal		324	49.92		449	56.62		
Abmormal		325	50.08		344	43.38	6.44	**0.010**
**EGFR**	222			347				
Mutant		109	49.10		154	44.81		
Nonmutant		113	50.90		192	55.19	1.146	0.284
**Surgery**	954			2261				
Yes		302	31.66		1154	51.04		
No		652	68.34		1107	48.96	81.61	**0.000**
**Radiotherapy**	954			2261				
Yes		249	26.10		472	20.88		
No		705	73.90		1789	79.12	10.53	**0.000**
**Chemotherapy**	954			2261				
Yes		632	66.25		1370	60.59		
No		322	33.75		891	39.41	9.13	**0.000**
**Targeted therapy**	954			2261				
Yes		81	8.49		129	5.71		
No		873	91.51		2132	94.29	8.52	**0.000**

The median age of the younger group was 40 years (range 19–45), while that of the older group was 61 years (range 46–88). The proportion of female patients was significantly higher in the younger group than in the older group (50.84% vs 34.85%, *P* < 0.001). The ratio of patients with a family history of cancer was higher in the younger group than in the older group (12.37% vs 6.32%, *P* < 0.001).

There were significant differences in the distribution of histological subtypes of lung cancer between the two groups (*P* < 0.001). Adenocarcinoma was the most common histological subtype in both groups, but the younger group had a higher proportion with the adenocarcinoma. Squamous cell carcinoma was more common in the older group than in the younger group (24.60% vs 10.05%). In the younger group, SCLC was the second most common pathological type, but it ranked third in the older group (24.67% vs 14.29%). There were no statistically significant differences in either tumour sites or subsites between the groups (Table 2).

The distribution of stages was significantly different between the two groups. The younger group had a higher proportion of patients with advanced-stage disease at presentation than the older group (65.46% vs 60.77%, *P* < 0.001). With regard to biomarkers, there were no significant differences in CEA and SCC in the two groups, but the proportion of patients with abnormal NSE was much higher in the younger group than in the older group (50.08% vs 43.38%, *P* = 0.01). Among the lung cancer patients who were tested for EGFR gene mutations, the mutation rate in the younger group was higher than that in the older group, but the difference between the two groups was not significant (49.10% vs 44.81%, *P* = 0.284). The resluts were shown as Table 2.

The proportions of patients who underwent surgery, radiotherapy, chemotherapy and targeted therapy information were also analysed. Significantly higher proportions of patients in the younger group received chemotherapy and targeted therapy; in contrast, significantly higher proportions of patients in the older group underwent surgery and radiation therapy (*P* < 0.001) (Table 2).

### Survival analysis

#### Epidemiological factors affecting survival in lung cancer patients

**Figure 2:**
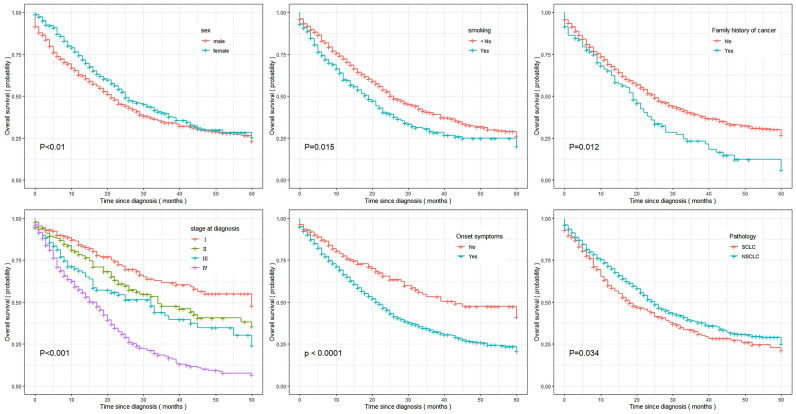
Estimate of five-year relative survival time for the young lung cancer patients.

In the hospital-based database, the median survival time for the entire population was 26.0 months, with survival durations of 23.0 months and 27 months in the younger group and older group, respectively. The 1-year, 3-year, and 5-year OS rates for the younger lung cancer patients were 68.58%, 36.86% and 27.14%, respectively. Among the older population, the rates were 75.11%, 41.83% and 30.82%, respectively. The survival rate in younger lung cancer patients was lower than that in those older than 45 years, with an HR of 0.829 (95% CI 0.747, 0.920). In the younger group, the median OS times were 21 months and 25 months for males and females, respectively. Female patients had a slightly better prognosis than males, and the difference was significant in univariate analysis (*P* = 0.002) (Fig. 2). The same results were observed in the group of older lung cancer patients and the entire population (Table 3).

**Table 3:** Univariate analysis of prognostic factors for lung cancer patients by age groups.

	≤ 45		> 45		Total	
Characteristic	median survival month (95%CI)	*P value*	median survival month (95%CI)	*P* value	median survival month (95%CI)	*P value*
Overall	23.0 (20.678,25.322)		27.0 (24.858,29.124)		26.0 (24.343,27.657)	0.001
**Sex**						
Male	21.0 (17.616,24.384)		24.0 (21.679,26.321)		23.0 (21.215,24.785)	
Female	25.0 (21.106,28.894)	**0.002**	38.0 (32.419,43.581)	** < 0.001**	32.0 (27.937,36.063)	< 0.001
**Family history**						
Yes	20.0 (19.709,23.291)		28.0 (25.620,30.380)		25.0 (21.674,28.326)	
No	25.0 (22.123,27.877)	**0.012**	31.0 (20.612,41.388)	0.408	26.0 (24.674,27.867)	0.284
**Smoking**						
Yes	19.0 (15.699,22.301)		24.0 (21.685,26.315)		23.0 (21.262,24.738)	
No	25.0 (21.507,28.493)	**0.001**	35.0 (30.038,39.692)	** < 0.001**	30.0 (26.724,33.276)	< 0.001
**Drinking**						
Yes	21.0 (16.014,25.986)		26.0 (22.970,29.030)		24.0 (21.469,26.531)	
No	25.0 (22.158,27.842	0.427	30.0 (26.424,33.576)	**0.007**	27.0 (24.826,29.174)	0.012
BMI						
< 24.0	23.0 (20.508,25.492)		26.0 (23.066,28.934)		25.0 (23.017,26.983)	
> 24.0	25.0 (20.931,29.069)	0.357	29.0 (25.952,32.048)	**0.043**	29.0 (26.198,31.802)	0.027
**Symptoms at diagnosis**						
Yes	22.0 (19.686,24.314)		26.0 (23.945,28.055)		24.0 (22.599,25.401)	
No	39.0 (17.972,60.028)	** < 0.001**	52.0 (41.754,62.246)	** < 0.001**	45.0 (38.106,51.894)	< 0.001
**Site**						
Left	23.0 (18.733,27.267)		25.0 (22.571,27.429)		24.0 (22.013,25.987)	
Right	25.0 (21.735,28.265)	0.443	31.0 (27.006,34.994)	**0.004**	28.0 (25.496,30.504)	0.007
**Subside**						
upper lobe	33.0 (25.807,40.193)		43.0 (35.924,50.076)		41.0 (31.265,40.735)	
Middle lobe	15.0 (4.024,25.976)		58.0 (44.756,71.244)		42.0 (22.468,37.532)	
Low lobe	37.0 (24.367,49.633)	0.174	44.0 (35.745,52.255)	0.504	42.0 (35.683,52.317)	0.773
**Cancer stage at diagnosis**						
I + II	63.0 (40.487,85.513)		–		79.0 (67.849,90.151)	
III + IV	18.0 (215.569,20.341)	** < 0.001**	19.0 (17.529,20.471)	** < 0.001**	18.0 (16.769,19.231)	< 0.001
**Pathology**						
SCLC	18.0 (12.791,23.209)		16.0 (13.669,18.331)		19.0 (16.391,21.609)	
NSCLC	25.0 (22.218,27.782)	**0.034**	31.0 (28.398,33.602)	** < 0.001**	27.0 (25.030,28.970)	< 0.001
**CEA**						
Normal	29.0 (23.543,34.457)		27.0 (23.208.30.792)		28.0 (24.507,31.493)	
Abmormal	19.0 (16.680,21.320)	** < 0.001**	21.0 (18.709.23.231)	** < 0.001**	20.0 (18.406,21.549)	< 0.001
**SCC**						
Normal	24.0 (20.467,27.533)		31.0 (27.388,34.612)		28.0 (25.076,30.924)	
Abmormal	18.0 (8.368,27.632)	**0.006**	20.0 (12.242,27.758)	**0.001**	20.0 (15.424,24.576)	< 0.001
NSE						
Normal	31.0 (23.192,38.808)		28.0 (24.052,31.948)		29.0 (25.321,32.679)	
Abmormal	17.0 (13.805,20.195)	** < 0.001**	20.0 (16.390,23.610)	** < 0.001**	19.0 (16.707,21.293)	< 0.001
**EGFR**						
Mutant	24.0 (8.882,39.118)		39.0 (16.171,61.829)		34.0 (26.573,41.427)	
Not mutant	29.0 (21.958,36.042)	0**.0**51	40.0 (30.651,49.349)	0.306	34.0 (20.139,47.861)	0.111
**Surgery**						
Yes	60.0 (41.879,78.121)		69.0 (63.190,74.810)		69.0 (61.807,76.193)	
No	18.0 (15.483,28.157)	** < 0.001**	18.0 (16.606,19.394)	** < 0.001**	18.0 (16.812,19.188)	< 0.001
**Radiotherapy**						
Yes	23.0 (18.494,27.506)		33.0 (29.594,36.406)		30.0 (27.607,32.393)	
No	24.0 (21.343,26.657)	0.213	20.0 (18.147,21.863)	** < 0.001**	21.0 (19.170,22.830)	< 0.001
**Chemotherapy**						
Yes	23.0 (20.444,25.556)		31.0 (24.720,37.280)		29.0 (24.600,33.400)	
No	23.0 (19.368,28.632)	0.497	27.0 (24.482,29.518)	0.222	26.0 (24.111,27.899)	0.094
**Targeted therapy**						
Yes	24.0 (21.301,26.699)		28.0 (25.551,30.449)		27.0 (25.151,28.849)	
No	21.0 (19.025,22.975)	0.474	26.0 (21.219,30.781)	0.436	23.0 (19.909,26.091)	0.207

Regardless of group, the prognosis for smokers was much lower than that for never smokers, and the survival time for patients with symptoms at diagnosis was much lower than that for patients without symptoms at diagnosis. The median survival time was shorter for young lung cancer patients with a family history of cancer than for those without a family history of cancer (20 m vs 25 m, *P* = 0.012) (Fig. 2, Table 3).

#### Clinical factors affecting survival in lung cancer patients

Aa the Table 3 shown that there was no difference in survival time in the younger group based on tumour location (*P* = 0.443). In contrast, there were significant differences in survival times in the older group according to tumour site (*P* = 0.004). Subsites were not significantly correlated with survival in any of the two groups (*P* = 0.174 and *P* = 0.504).

There were significant differences in survival according to stage in the two groups. The five-year survival rates in the younger group and the older group were as follows: 52.55% and 67.41% with stage I disease (*P* < 0.01), 38.12% and 49.23% with stage II disease (*P* = 0.025), 30.31% and 24.58% with stage III disease (*P* = 0.945), and 7.05% and 7.71% with stage IV disease (*P* = 0.451), respectively. The median survival times for patients with SCLC and NSCLC were 18.0 months and 25.0 months in the younger group and 16.0 months and 31.0 months in the older group, respectively. There were significant differences in the survival times by pathology in both age groups (Table 3).

In the younger group, patients with EGFR mutations had a median survival time of 29.0 months, while those without EGFR mutations had a median survival time of 24 months; the difference was not significant (*P* = 0.051). The same result was found in the older group. With regard to CEA, NSE and SCC, the survival time in the patients with abnormal levels was much lower than that in those with normal levels in the both groups.

Lung cancer patients who underwent surgery had a clearly better prognosis than the younger without surgery in the groups. Young patients who underwent radiotherapy didn’t have a better survival outcome than patients without radiotherapy. There was no significant difference in the groups based on whether they underwent chemotherapy or targeted therapy (Table 3).

### Cox regression analysis of the prognostic factors in young lung cancer patients

**Figure 3:**
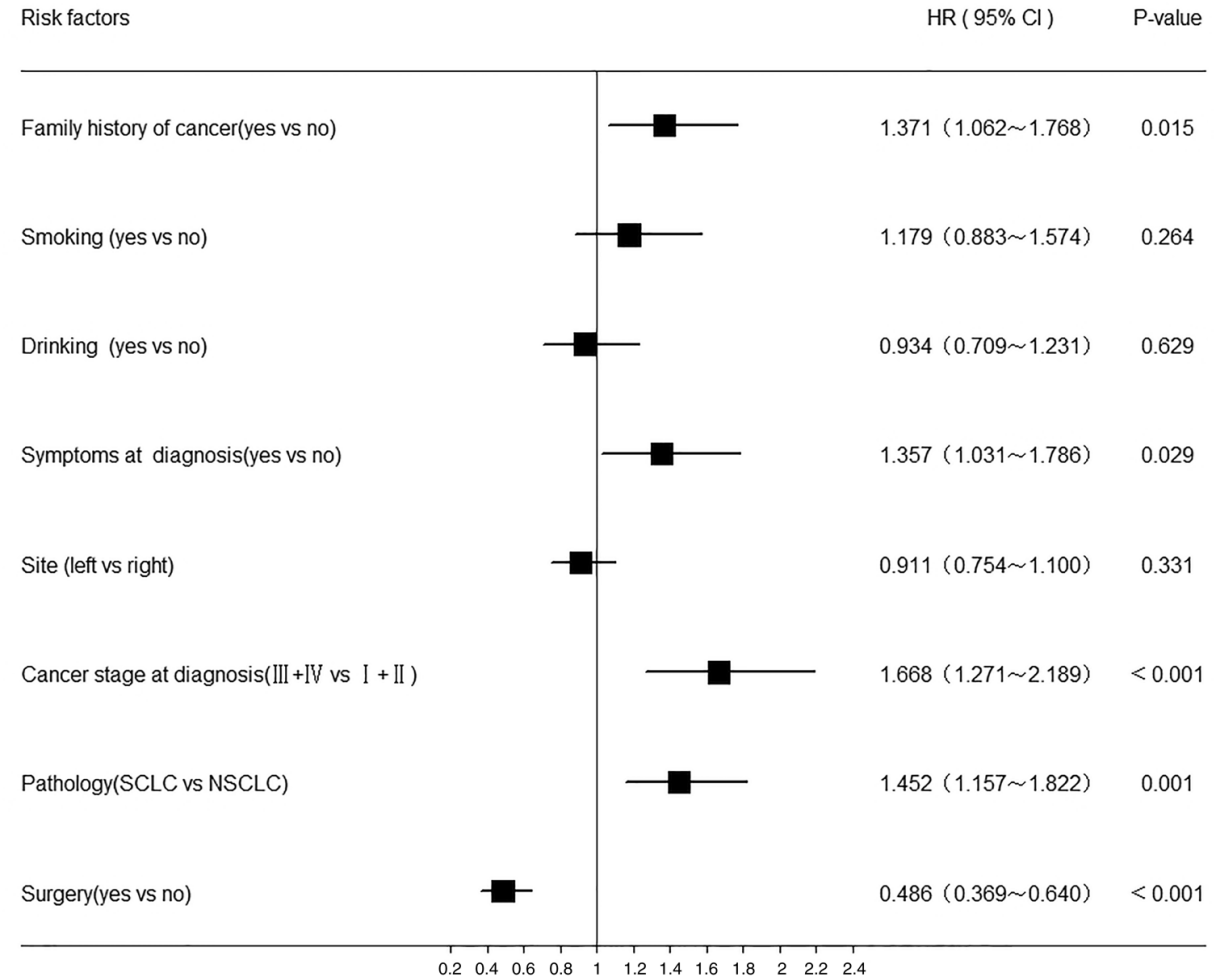
HR of risk factors associated with lung cancer patients younger than 45 years old by Multivariate Cox regression analysis. Analyses were adjusted for gender, family history of cancer, smoking, drinking, onset symptoms, site, cancer stage at diagnosis, pathology and surgery.

Most patients had missing values for CEA, NSE, SCC, EGFR and subsite; therefore, those variables were excluded from the Cox model. The results are shown in Fig. 3. According to the Cox regression analysis of the younger lung cancer patients, there were five independent factors that affected prognosis, namely, a family history of cancer, symptoms at diagnosis, cancer stage at diagnosis, pathological subtype and surgery. A significantly higher risk of mortality was identified in patients with a family history of cancer (HR = 1.371, 95% CI 1.062, 1.768) and symptoms at diagnosis (HR = 1.357, 95% CI 1.031, 1.786). Advanced stage at diagnosis was also a significant risk factor, with an HR of 1.668 (95% CI 1.271, 2.18 +). Surgery (HR = 0.486, 95% CI 0.369, 0.640) was associated with a significantly reduced risk of mortality in younger lung cancer patients. Compared with patients with NSCLC, those with SCLC had an increased risk of mortality (HR = 1.452, 95% CI 1.157, 1.822).

## Discussion

Several previous studies have compared clinical features and prognoses between younger and older lung cancer patients. However, the broad category of younger patients encompasses a wide range of subgroups, and different age subgroups of younger patients might have distinctive clinical characteristics and survival outcomes. Therefore, in the context of the dramatic increase in the incidence of lung cancer in the population aged 45 years and older and the relative rarity of patients younger than 45 years old in Hebei Province, according to the cancer registry database, this study was performed to analyse the clinicopathologic characteristics and survival outcomes in the latter population. The results showed that the mortality rate has increased in younger lung cancer patients over the last 40 years, and a family history of cancer, symptoms at diagnosis, advanced stage at diagnosis, adenocarcinoma and no surgical treatment were independent negative prognostic factors in younger lung cancer patients in Hebei Province. As this issue has seldom been addressed, the results of the present study may improve the clinical understanding of the characteristics of younger lung cancer patients.

According to the cancer registry database, Hebei Province has a heavy lung cancer burden, and the incidence has clearly increased over the past 40 years^31^. The ASMR were 10.69 per 100,000, 23.17 per 100,000, 26.64 per 100,000 and 28.15 per 100,000 in 1973–1975, 1990–1992, 2004–2005 and 2010–2015, respectively, with an overall increase of 163.33%^32^. A similar trend was shown in younger patients, with a 30% increase from the 1970s to the 2010s. In addition, there was an obvious increasing trend in the median age at death over the last 40 years, from 38.80 years to 40.88 years, among younger lung cancer patients, which is consistent with the overall trend in age-specific mortality in Hebei Province^31^. This study showed that in the younger age group, the mortality rate was higher in males than in females, according to the data from both the population- and hospital-based databases. Moreover, the PDR was higher in females than in males, according to the data from both the population- and hospital-based databases. This indicates the reliability and stability of the data from these two sources.

In the hospital-based database, we identified several interesting characteristics of younger patients with lung cancer. The proportion of females in the younger group was 50.84%, which was much higher than that in the older group with 34.85%; in addition, in the younger patients, the proportion of females was slightly higher than that of males, which was consistent with some previous related studies^33–36^. Adenocarcinoma was the most common histological subtype in the younger patients, accounting for 65.27%, which was higher than the proportion in the older group. SCLC and squamous cell carcinoma were the second and third most common subtypes in the younger group, respectively, which was similar to the results in several articles from China^37–39^, but inconsistent with the results from other countries. squamous cell carcinoma and SCLC ranked the second and third most common subtype in the younger group^40–42^. A relatively high percentage of patients had advanced-stage disease at diagnosis in our study (greater than 50% of all patients), which was consistent with previous studies^12,43^.

The correlation between age and genetic abnormalities is controversial. According to several studies, there is a trend towards a greater proportion of older patients harbouring EGFR mutations^44,45^. However, other studies reported the opposite result^46,47^. Sacher et al. found that younger patients had higher frequencies of driver mutations than older patients in a Caucasian population (EGFR mutations: 32% vs 23%)^13^. In another study by Scarpino et al., they mentioned that the frequency of EGFR driver mutations was 23% in younger patients, which was higher than the 16% in older patients^48^. In our current study, among the lung cancer patients who underwent testing for EGFR mutations, 49.10% had mutations in the younger group, and 44.81% had mutations in the older group; the difference was not significant, which was consistent with a recent study in Chinese patients^46^. CEA is one of the most widely used tumour markers, and elevated CEA expression is often observed in patients with lung cancer, especially those with adenocarcinoma^49,50^. SCC has been confirmed to be closely related to the prognosis of lung cancer. Sone studies have suggested that the level of SCC in exhaled breath condensate might serve as a marker that could be used to achieve an early diagnosis of lung cancer^51,52^. NSE is currently the most reliable tumour marker, and it is used for the diagnosis, prognostic prediction and follow-up monitoring of small-cell lung cancer^53^. However, the expression levels of serum biomarkers have rarely been reported in younger lung cancer patients in previous studies. Therefore, our current study investigated the relationships between these three tumour markers and patient age. There were no significant differences between the two age groups with regard to CEA and SCC. Interestingly, the level of expression of NSE was much higher in the younger group than that in the older group, which was consistent with the study by Yu et al. although the cut-off age in that study was 60 years old^51^.

In previous studies, there was a significant difference in the smoking rate between younger and older lung cancer patients. In our study, 28.83% of the younger group smoked, which is lower than the proportions reported internationally, such as in Japan (47.4–66%) and the USA (80–93%)^12,20,41,42,54–56^. Nevertheless, the proportion of smokers in our study is consistent with those reported in Liu's and Hou's studies, with the proportions of lung cancer patients who were smokers reported as 28.40% and 28.81% in China, respectively^34,46^, in contrast, the proportion in our study was higher than that in the study by Laurence et al., which reported that approximately 14.0% of the lung cancer patients younger than 40 years in France and Peru were smokers^23,57^. A family history of cancer was regarded as a surrogate for a genetic predisposition for lung cancer in some previous studies^24^. The proportions of patients with a family history of cancer differed between younger and older patients. Our current study found a relatively high proportion of family history of cancer in younger lung cancer patients, this finding is consistent with the findings of previous studies. A Polish study suggested that patients younger than 50 years old were relatively more likely than their older counterparts to have had a mother who had had lung cancer (4.7% vs 3.0%; P < 0.001) and to have had a father who had had lung cancer (7.6% vs 4.1%, P < 0.001)^58^. Another study by Laurence et al. reported that the relatives of 25% of the younger patients who had a family history of lung cancer had developed the disease before they were 45 years old, compared to less than 5% in the old group^23^. One study by Abbasowa et al. reported that the proportions of the younger and older groups of patients who had a family history of cancer were 44.8% and 36.0%, respectively, and the difference was statistically significant^59^. In contrast, other studies by Wei et al. and Anna et al. reported that although the proportion in younger individuals was slightly higher than that in older individuals, the difference was not significant^37,54^. Symptoms, including cough, thoracic pain and tightness, at diagnosis are common. However, a previous study reported that younger patients were more frequently asymptomatic at diagnosis, while older patients were significantly more likely to have thoracic pain, cough and fatigue (p < 0.01)^60^, The proportions of younger and older patients with symptoms at diagnosis according to the data from the hospital-based database in Hebei Province were roughly similar to those in other countries.

According to the literature, smoking is regarded as a major and most well-established risk factor and independent prognostic factor for lung cancer^61^. A previous study suggested that an estimated 75.04% of lung cancer-related deaths in men and 18.35% in women are attributable to tobacco use in China^62^. In agreement with these findings, our study found that the survival time for patients who smoked was shorter than that for patients who had never smoked in the younger group. This finding was consistent with the study by Ayesha et al., which reported that a Kaplan–Meier survival analysis showed that smoking status significantly impacted survival^63^. A family history of cancer was found to be an independent prognostic factor in younger lung cancer patients in the current study. This finding has already been reported in some studies, such as that by Ganti et al. Multivariate Cox regression showed that that patients with a family history of cancer had an HR for mortality of 1.371 (95% CI 1.062, 1.768) compared to those without a family history of cancer, which was consistent with a US study that found an adjusted hazard ratio for mortality of 1.65 in the group with a family history of lung cancer compared with the group without such a family history^64^. A previous study in Japan demonstrated that inherited genetic susceptibility, as reflected in the family history, may contribute to the development of lung cancer^24^. However, the adjusted hazard ratio for death was not significant in the older group of patients, which may be because relatively few patients had a family history of cancer in the older group.

There have been conflicting findings regarding about the survival and prognosis of younger patients with lung cancer. Perhaps due to the differences in the cut-off age or use of surgery in the various populations of younger lung cancer patients, the findings have varied across studies^13,37,42,43,54,57,63,65,66^. In our study, the survival time for younger patients was relatively worse than that for older patients. There are some explanations for this finding. The clinical stage at diagnosis is well known to be the most important predictor of survival, and the proportion of patients with advanced-stage disease was higher in the younger group than in the older group (P < 0.001). It has been suggested that lung cancer tends to be more aggressive in nature, to have a more rapid development or to be the result of underlying genetic predispositions in younger patients^33,43,67^. Younger patients with lung adenocarcinoma, especially those with advanced-stage disease, did not have significantly better overall survival than older patients despite undergoing more aggressive treatment^11^. On the other hand, because younger patients are more frequently asymptomatic at diagnosis, it is possible that younger patients are less likely to suspect cancer, which may result in diagnostic and surgical delays, resulting in disease progression^63^. Other factors (e.g., late diagnosis, disease awareness, and financial challenges) represent potential contributors to or alternative explanations of this phenomenon^13^.

The conflicting results suggest that there is considerable heterogeneity among younger adults with lung cancer. Large-scale, multicenter, and nationally representative data are urgently needed to fully explore the characteristics and prognosis of lung cancer in younger patients and to determine the most effective interventions^34^. For younger individuals, especially individuals who have a family history of cancer, a history of smoking, symptoms (such as cough, chest pain, hemoptysis) or abnormal biomarker levels, regular screening for lung cancer is recommended, and it is necessary to seek medical assistance promptly.

## Limitations

In interpreting these findings, several limitations inherent to this study must be considered. The older lung cancer patients included in this study were selected randomly from the entire age group, introducing the possibility of referral bias. The second limitation is that only a relatively small number of patients underwent EGFR mutation analysis because at the time of this study, this test was not mandatory in China. Furthermore, due to the retrospective nature of the data collection, we were not able to analyse potentially prognostic variables, such as environmental factors, occupational exposure to carcinogens, other gene mutations, family financial status, and novel cancer therapies, which might be of prognostic value in lung cancer patients.

## Conclusion

In conclusion, our study indicates that younger patients with lung cancer have distinctive characteristics, including a higher percentage of female patients, family history of cancer, more adenocarcinoma and more advanced stage at diagnosis. We noticed that younger lung cancer patients appear to trend a worse prognosis. A family history of cancer, symptoms at diagnosis, pathology, stage at diagnosis and surgery were confirmed as independent prognostic factors in younger lung cancer patients. Therefore, for younger individuals, especially the individuals who have a family history of cancer, smoking, symptoms (such as cough, chest pain, haemoptysis) or abnormal biomarker levels, regular screening for lung cancer is recommended. In addition, considering the conflicting results with regard to younger patients, large-scale, multicentre, and nationally representative data are urgently needed to fully explore characteristics and prognosis of lung cancer in younger patients and to determine the most effective interventions.
